# Reliability of body composition assessment using A-mode ultrasound in a heterogeneous sample

**DOI:** 10.1038/s41430-020-00743-y

**Published:** 2020-09-11

**Authors:** Monica Miclos-Balica, Paul Muntean, Falk Schick, Horia G. Haragus, Bogdan Glisici, Vasile Pupazan, Adrian Neagu, Monica Neagu

**Affiliations:** 1 Center for Modeling Biological Systems and Data Analysis, Department of Functional Sciences, Victor Babeş University of Medicine and Pharmacy, Timişoara, Romania; 2 Department of Orthopedics, Victor Babeş University of Medicine and Pharmacy, Timişoara, Romania; 3 Department of Physics & Astronomy, University of Missouri, Columbia, MO USA

**Keywords:** Risk factors, Techniques and instrumentation

## Abstract

**Background/Objectives:**

Several studies have addressed the validity of ultrasound (US) for body composition assessment, but few have evaluated its reliability. This study aimed to determine the reliability of percent body fat (%BF) estimates using A-mode US in a heterogeneous sample.

**Subjects/Methods:**

A group of 144 healthy adults (81 men and 63 women), 30.4 (10.1) years (mean (SD)), BMI 24.6 (4.7) kg/m^2^, completed 6 consecutive measurements of the subcutaneous fat layer thickness at 8 anatomical sites. The measurements were done, alternatively, by two testers, using a BodyMetrix™ instrument. To compute %BF, 4 formulas from the BodyView™ software were applied: 7-sites Jackson and Pollock, 3-sites Jackson and Pollock, 3-sites Pollock, and 1-point biceps.

**Results:**

The formula with the most anatomic sites provided the best reliability quantified by the following measures: intraclass correlation coefficient (ICC) = 0.979 for Tester 1 (T1) and 0.985 for T2, technical error of measurement (TEM) = 1.07% BF for T1 and 0.89% BF for T2, and minimal detectable change (MDC) = 2.95% BF for T1, and 2.47% BF for T2. The intertester bias was −0.5% BF, whereas the intertester ICC was 0.972. The intertester MDC was 3.43% BF for the entire sample, 3.24% BF for men, and 3.65% BF for women.

**Conclusions:**

A-mode US is highly reliable for %BF assessments, but it is more precise for men than for women. Examiner performance is a source of variability that needs to be mitigated to further improve the precision of this technique.

## Introduction

The evaluation of human body composition is important in sports medicine [1] and in clinical disciplines in which the treatment plan includes body weight management. For example, the therapy of obese patients seeks to reduce their body fat mass (FM) while minimizing the loss of fat-free mass (FFM) [2]. The efficacy of such a therapy can be monitored using techniques of body composition analysis [3]. While laboratory methods, such as dual energy x-ray absorptiometry (DXA), magnetic resonance imaging, underwater weighing (UWW), and air displacement plethysmography (ADP) are considered to be accurate (valid), they require expensive equipment and adequate space [1, 4, 5]. Field methods, such as anthropometry, bioelectrical impedance analysis (BIA), and ultrasound (US) involve relatively inexpensive, portable instruments. They are suitable for bedside or event-site use [1], but their validity and reliability needs to be tested for various populations [6].

An increasing body of evidence indicates that US might become a powerful technique of body composition assessment as new hardware and software is being developed for this purpose [7, 8]. Several studies have assessed the validity of US, yielding mixed results. For 89 healthy adults, US measurements combined with anthropometry provided percent body fat (%BF = FM/(FM + FFM) × 100%) estimates in good agreement with DXA, but with significant bias compared to ADP and BIA [9]. US assessments of %BF were accurate also in a sample of 93 athletes [10]. Nevertheless, using a different device and various prediction formulas for computing %BF, two other groups found significant differences between US and DXA [11, 12]. In a study of 70 high school wrestlers, FFM values measured via US were not statistically different from values obtained by UWW; moreover, a Bland–Altman analysis indicated negligible bias between US and UWW [13]. A cross-validation study of US, BIA, and ADP found high Pearson correlations: 0.862 between US and BIA, and 0.872 between US and ADP [14]. Compared with a three-compartment model, US underestimated %BF by 4.7% and overestimated FFM by 4.4 kg in a sample of 47 overweight and obese subjects [15]. In a study of 45 elite athletes, US overestimated %BF by about 3% in comparison to ADP [6]. Prediction equations developed for Brazilian adults enabled US measurements of %BF in good agreement with ADP, leading to a bias of 0.5% for men and 0.1% for women [16]. Also, a study of 31 normal weight adults reported no bias between US and ADP [17]. The validity of US was confirmed for measurements of the subcutaneous adipose tissue layer thickness. The accuracy of brightness (B)-mode measurements was <0.5 mm on excised pig tissues [18], whereas on cadavers the accuracy was <1 mm for both amplitude (A)-mode and B-mode US at several anatomic sites commonly used in skinfold thickness measurements [19].

Although it is vital to further establish the validity of US as a tool of body composition analysis, in certain cases its reliability is even more important (e.g., when tracking the progress of a treatment program over time). The reliability of US for %BF measurements has been tested by several studies [6, 15, 17, 20, 21], albeit on relatively small, homogeneous samples. Moreover, the reliability studies published so far focused on just a few prediction formulas, although it is known that the choice of formula affects both validity [11, 12] and reliability [20]. Therefore, the present study was conducted to examine the reliability of US for %BF assessments using 4 prediction formulas in a heterogeneous sample and determine whether this reliability depends on the subject’s gender.

It has been recommended that in a reliability study at least two examiners should perform triplicate trials for at least 50 subjects per condition (e.g., for each gender) [22], and measures of reliability should include both a relative and an absolute measure [23]. Our study was designed accordingly.

## Subjects and methods

### Subjects

Participants were recruited via social networks and flyers posted in the local community. Clinically healthy adults, aged 18–70 years, were included in the study upon providing written informed consent. The resulting sample was composed of 144 volunteers (81 men and 63 women). Conducted according to the Declaration of Helsinki, this study was approved by our institutional Committee of Research Ethics. Table 1 describes the participants in terms of age, height, body mass (BM) and body mass index (BMI)—body mass (kg) divided by height squared (m^2^). Data are reported as the mean followed by the standard deviation (SD) enclosed in brackets; the range of values is given by listing the minimum (Min.) and the maximum (Max.).

**Table 1:** Characteristics of the study population.

	All (*n* = 144)			Men (*n* = 81)			Women (*n* = 63)		
	Mean (SD)	Min.	Max.	Mean (SD)	Min.	Max.	Mean (SD)	Min.	Max.
Age (y)	30.4 (10.1)	19	66	29.8 (9.2)	20	66	31.1 (11.1)	19	62
Height (m)	1.72 (0.1)	1.51	1.96	1.78 (0.07)	1.63	1.96	1.64 (0.06)	1.51	1.80
BM (kg)	72.8 (15.8)	37.9	120.2	80.2 (13.7)	55.0	120.2	63.2 (13.1)	37.9	108.8
BMI (kg/m^2^)	24.6 (4.7)	16.6	45.0	25.2 (3.9)	17.0	40.3	23.7 (5.4)	16.6	45.0

### A-mode ultrasound measurements

Measurements were done using a BodyMetrix™ BX2000 ultrasound instrument (IntelaMetrix, Livermore, CA, USA)—called BodyMetrix hereafter—working in A-mode at a frequency of 2.5 MHz. We measured BM to the nearest 0.01 kg using the scale connected to a BOD POD Gold Standard Body Composition Tracking System (COSMED USA, Concord, CA, USA). Scale calibration was carried out daily. Height was measured to the nearest 1 mm using a wall mounted tape measure (GIMA 27335, GIMA, Gessate, Italy). We created a new client profile for each participant in the BodyView™ software (v5.7.11043) specifying name, age, gender, height, weight, and athletic type. Lean and normal weight subjects (BMI < 25) were deemed “Athletic”, whereas overweight and obese subjects were designated as “Non-Athletic”. Our sample did not include elite athletes. We followed the manufacturer’s recommendations [24] for making A-mode US measurements of the subcutaneous adipose tissue layer thickness at 8 anatomical locations: biceps, triceps, chest, scapula, axilla, waist, hip, and thigh. The objective of this study was to evaluate the precision of routine assessments of body composition. Therefore, in evaluating the subcutaneous fat thickness, we relied entirely on the BodyView software’s automatic algorithm for spotting the fat-muscle interface. The examiner placed a pea-sized amount of ultrasound conductive gel on the transducer head, placed it in contact with the skin, and for the duration of the measurement (4–8 s) she/he slid the transducer about 0.5 cm above and below the chosen site, while maintaining a slight, steady inward force on the transducer—so as not to deform the underlying tissue. Transducer movement assured a local averaging (smoothing) of the recorded signal.

Two testers, with about 1 year of experience, took all measurements in triplicate. Before testing a new subject, they flipped a coin to decide which of them would take the first set of data while the other would record the data. After each set, the testers swapped their roles until three sets of data were obtained by each. The results of the two testers were recorded in different portions of a spreadsheet, making it difficult for the recorder to compare a new result with her/his own assessment of the same anatomic location.

Percent body fat was assessed using 4 formulas implemented in BodyView: 7-sites Jackson and Pollock (JP7), 3-sites Jackson and Pollock (JP3), 3-sites Pollock (P3), and 1-point biceps (BIC). Each set of measurements started with BIC; then, JP7 was selected and the corresponding sites were measured. To compute %BF via JP3 and P3, the thicknesses recorded during the JP7 assessment were fed manually into BodyView.

### Statistical analysis

The data was analyzed using the Statistics Toolbox from MATLAB 7.13 (The MathWorks, Natick, MA, USA). Statistical significance was set at *P* ≤ 0.05.

We used Bland–Altman (BA) analysis to characterize the agreement between successive readings performed by one tester (intratester agreement) or between readings performed by different testers (intertester agreement). In BA plots, the differences, *d*_*i*_, of measured data pairs are represented versus their mean (the index *i* labels subjects, *i* = 1, 2, …, *n*, where *n* is the sample size). Shown are also the bias, defined as the mean value of the differences, $\overline d = \frac{1}{n}\left( {\mathop {\sum}\nolimits_{i = 1}^n {d_i} } \right)$, and the 95% limits of agreement, $\overline d \pm 1.96\,{\mathrm{SD}}_{\mathrm{d}}$, where SD_d_ denotes the standard deviation of the differences [25]. Intra - and intertester agreement was characterized also in terms of the difference between the upper limit of agreement (ULA) and the lower limit of agreement (LLA); ULA − LLA = 2 × (ULA − Bias).

We computed one relative measure of reliability, the intraclass correlation coefficient (ICC) [23, 26], and 3 absolute measures of reliability: the technical error of measurement (TEM), the standard error of measurement (SEM), and the minimal detectable change (MDC) [23, 27–29]. TEM was computed as $\sqrt {\frac{1}{{2n}}\mathop {\sum}\nolimits_{i = 1}^n {d_i^2} }$. ICC was obtained from the two-way random effects model, denoted as ICC(2,1) [30]. SEM was computed as ${\mathrm{SD}}\sqrt {1 - {\mathrm{ICC}}}$, where SD denotes the standard deviation of all 2*n* values obtained in pairs of trials conducted on *n* subjects [23]. MDC was calculated as $1.96 \cdot \sqrt 2 \cdot {\mathrm{SEM}}$, where 1.96 is the two-sided *z*-score that corresponds to the 95% confidence level and $\sqrt 2$ accounts for the variance of two measurements [28, 29].

## Results

**Fig. 1:**
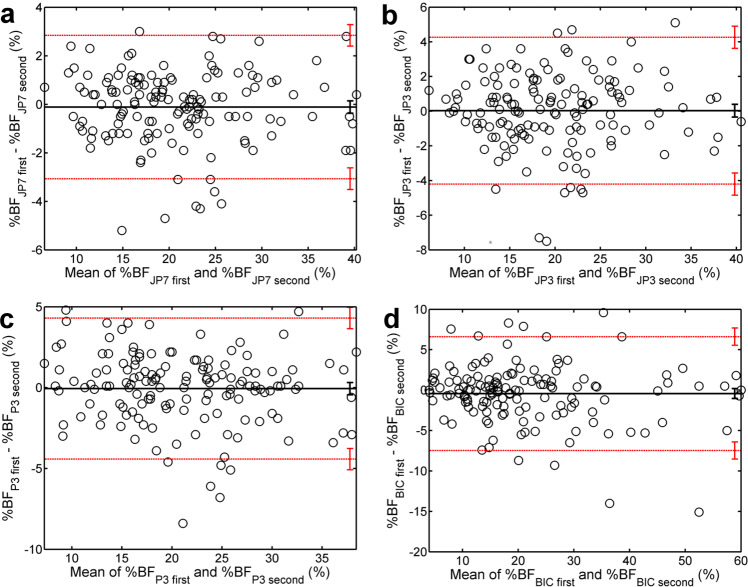
BA analysis of intratester agreement. BA plots of differences vs. means of the first and second assessment of %BF by Tester 1 using A-mode ultrasound and four different formulas: **a** 7-sites Jackson and Pollock (JP7), **b** 3-sites Jackson and Pollock (JP3), **c** 3-sites Pollock (P3), and **d** 1-point biceps (BIC). The thick horizontal line depicts the bias, whereas the thin dotted lines depict the 95% limits of agreement. The vertical segments on the right represent the 95% confidence intervals (CI) of the corresponding quantities.

Figure 1 illustrates the agreement between two sets of ultrasound measurements performed by Tester 1. Similar plots were obtained for Tester 2 (see Supplementary Material, Fig. S1).

In Fig. 1, experimental points are evenly distributed around the solid line that represents the bias—i.e., the intratester agreement is unaffected by the subject’s adiposity. In each panel, zero belongs to the 95% confidence interval (CI) of the bias. The width of the 95% interval of agreement, ULA–LLA, is smallest in panel (a), indicating that the JP7 formula assures the best intratester reliability, being followed by JP3 and P3 on the same footing (panels (b) and (c)), and by BIC (panel (d)). Similar results were obtained, for both testers, when the first reading was compared with the third (1–3) and when the second reading was compared with the third (2–3) (Table S1).

Table 2 presents TEM, SEM, MDC, and ICC for %BF values obtained in pairs of consecutive trials. For Tester 1 (T1), the reliability parameters were the best for the %BF values recorded in the second and third trial (pair 2–3) for the JP7, JP3, and P3 formulas, but not for the BIC formula. For T2, the best reliability was observed for pair 1–3.

**Table 2:** Statistical parameters of intratester reliability of %BF assessments by 4 different anthropometric formulas (JP7, JP3, P3, and BIC), computed for three pairs of measurements (1–2, 1–3, and 2–3) performed by two testers.

Formula	Pair	Tester 1				Tester 2			
		TEM^a^	SEM	MDC	ICC^b^	TEM	SEM	MDC	ICC
JP7	1–2	1.065	1.063	2.947	0.9792	0.893	0.892	2.472	0.9854
	1–3	1.213	1.211	3.356	0.9726	0.916	0.915	2.535	0.9847
	2–3	0.983	0.981	2.719	0.9821	0.969	0.968	2.683	0.9825
JP3	1–2	1.522	1.52	4.212	0.9542	1.417	1.415	3.921	0.9598
	1–3	1.794	1.791	4.964	0.9341	1.391	1.388	3.848	0.9613
	2–3	1.473	1.47	4.075	0.9556	1.521	1.519	4.21	0.9525
P3	1–2	1.569	1.567	4.343	0.9552	1.282	1.28	3.549	0.9699
	1–3	1.587	1.584	4.391	0.9522	1.233	1.231	3.413	0.9718
	2–3	1.488	1.486	4.118	0.9598	1.395	1.392	3.859	0.9642
BIC	1–2	2.548	2.544	7.051	0.9642	2.555	2.551	7.071	0.9632
	1–3	1.778	1.775	4.92	0.9818	2.192	2.189	6.067	0.9734
	2–3	2.507	2.503	6.937	0.9653	2.556	2.551	7.072	0.9649

^a^TEM, SEM, and MDC are expressed in the same units as the measured quantity (%BF).

^b^ICC stands for ICC (2,1)—the 2-way random model, single score intraclass correlation coefficient; it is dimensionless, ranging from 0 to 1—the higher, the better [22].

Table 2 and S1 reveal no systematic differences between pairs. Therefore, the remainder of this paper deals with a single pair: (i) the first two trials of both testers when evaluating intratester reliability, and (ii) the first trial of T1 and the third trial of T2 when evaluating intertester reliability. The latter pair was the most separated in time, making it the least likely that the two testers have influenced each other.

Table 3 describes the impact of gender on the reliability of US for %BF measurements. The relative reliability was excellent for both testers [26]—ICC > 0.9 for all measurements, except for women assessed by JP3. Nevertheless, in most cases, T2 was more reliable than T1.

**Table 3:** Intra- and intertester reliability evaluated for the entire sample (*n* = 144), for men (*n* = 81), and for women (*n* = 63). Intratester reliability was assessed from the first pair of trials, whereas intertester reliability was evaluated from the values recorded in the first trial of Tester 1 and the third trial of Tester 2.

Formula	Subjects	Tester 1				Tester 2				Intertester			
		TEM^a^	SEM	MDC	ICC^b^	TEM	SEM	MDC	ICC	TEM	SEM	MDC	ICC
**JP7**	All	1.065	1.063	2.947	0.9792	0.893	0.892	2.472	0.9854	1.242	1.239	3.434	0.9715
	Men	0.957	0.954	2.644	0.9589	0.836	0.833	2.309	0.9704	1.174	1.169	3.241	0.9389
	Women	1.19	1.186	3.286	0.9634	0.963	0.959	2.657	0.9719	1.324	1.318	3.653	0.9534
**JP3**	All	1.522	1.52	4.212	0.9542	1.417	1.415	3.921	0.9598	1.759	1.755	4.865	0.9378
	Men	1.252	1.248	3.46	0.9437	1.295	1.29	3.577	0.9401	1.592	1.584	4.392	0.9081
	Women	1.811	1.805	5.004	0.8984	1.56	1.555	4.31	0.9191	1.953	1.946	5.395	0.8829

^a^TEM, SEM, and MDC are expressed in the same units as the measured quantity (%BF).

^b^ICC denotes ICC (2,1).

TEM, SEM, and MDC were smaller for men than for women (Table 3); ICC, on the other hand, was slightly higher for women than for men when the JP7 formula was applied, whereas for JP3 the situation was opposite. The gender dependence of intratester reliability is illustrated also by Figs. S2 and S3.

**Fig. 2:**
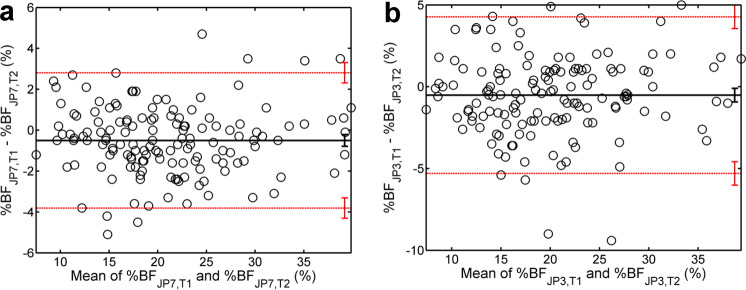
BA plots of intertester agreement. BA analysis of intertester agreement between %BF measurements based on (**a**) JP7 and (**b**) JP3. Each plot represents differences vs. means of the first reading of Tester 1 (T1) and the third reading of Tester 2 (T2). Notations are explained in the caption of Fig. 1.

We also applied the BA method to characterize the intertester reliability of %BF measurements via US (Fig. 2). The bias was −0.50% for the JP7 formula and −0.52% for the JP3 formula; in neither case has zero been part of the 95% CI of the bias. The width of the 95% interval of agreement was 6.61% for JP7 and 9.57% for JP3; these were larger than those corresponding to intratester agreement (Table S1). The intertester BA plots derived from the P3 and BIC formulas are shown in Fig. S4.

We hypothesized that the intertester bias originated from an underestimation of the subcutaneous adipose tissue layer thicknesses by T1 as compared to T2. To test this hypothesis, we computed S7—the sum of the 7 adipose layer thicknesses involved in the JP7 formula [31, 32]—and performed a paired t-test to decide whether the difference between the means of S7 recorded by the two testers is significantly different from zero. For the entire sample, the average of S7 computed from all $3n$ measurements performed by T1 was 75.6 mm, whereas the corresponding quantity recorded by T2 was 78.0 mm; their difference, −2.4 mm, was significantly different from zero (*P* = 7.7 × 10^−6^), with a 95% CI [−3.3, −1.3] mm. This underestimation was more pronounced for women (−3.0 mm; 95% CI [−4.7, −1.3] mm; *P* = 5.4 × 10^−4^) than for men (−1.8 mm; 95% CI [−3.0, −0.6] mm; *P* = 4.5 × 10^−3^), leading to a larger intertester bias for women (Fig. S5).

## Discussion

This work evaluated the intra- and intertester reliability of %BF assessments via A-mode US. To our knowledge, this is the first study of learning effects in the context of this technique and the first to evaluate its reliability in a heterogeneous sample of more than 50 subjects of each gender.

To check for learning effects on the measurement error, we analyzed consecutive pairs of trials [22]—first and second (1–2), first and third (1–3), second and third (2–3). The indices of precision derived from different pairs displayed no clear trend. Tester 1 (T1) had the best precision for pair 2–3 (as expected in the presence of learning effects), but only for the JP7, JP3, and P3 formulas. T2 was most precise for pair 1–2 with JP7 and for pair 1–3 otherwise. It seems safe to conclude that learning effects are absent in %BF estimates via A-mode US.

A change in a measured quantity is deemed to be real if it exceeds the MDC (i.e., MDC is the smallest change in score that is not due to measurement error) [29]. For a sample of college students [20], Loenneke et al. reported MDC values of 5.6% BF for JP3 and 2.8% BF for BIC. Unfortunately, our work did not confirm the superiority of the simplest formula: BIC gave the largest intratester MDCs, of about 7% BF. Our study suggests that the larger is the number of sites involved in a formula, the higher is the reliability of %BF estimates (Table 2). For JP7, MDC was 3% BF (2.6% for men and 3.3% for women) (Table 3). Hence, US is suitable to track moderate changes in body composition. The tracking becomes less precise if different examiners are involved, as shown by the intertester MDC = 3.4% BF (3.2% for men and 3.7% for women).

For JP7, the intratester TEM was similar to that of ADP [33], which suggests that the BodyMetrix is as reliable as the BOD POD.

Smith-Ryan et al. [15] evaluated the intratester reliability of the BodyMetrix instrument and the JP7 formula for obese and overweight subjects. They conducted two trials for each subject 24–72 h apart. Their relative consistency (ICC = 0.98) was similar to ours, but their typical error (SEM = 2.2% BF) was larger. This discrepancy might stem from sample differences, although our BA analysis did not indicate less reliability at high adiposity.

Using BodyMetrix with the JP3 formula, Hendrickson et al. [17] have found a test-retest ICC of 0.87 for rater 1 and 0.80 for rater 2, whereas the inter-rater ICC was 0.87. It is important to keep in mind that ICC normalizes the measurement error to sample heterogeneity; for the same trial-to-trial consistency, ICC is large when between-subjects variability is high [23]. Our sample (BMI = 24.6 (4.7) kg/m^2^) was more heterogeneous than theirs (BMI = 23.9 (3.0) kg/m^2^) [17], explaining the lower ICCs observed by these authors.

The best reliability indices reported in the literature for the BodyMetrix instrument were obtained by Wagner et al. [6] using the JP3 formula in a sample of 22 male and 23 female athletes (BMI = 24.1 (2.4) kg/m^2^). Their test-retest ICC was 0.996 for technician 1 and 0.993 for technician 2, whereas the intertester ICC was 0.987; MDC was 1.3% BF for technician 1 and 1.8% BF for technician 2. In comparison with earlier works [15, 20], Wagner et al. attributed their superior reliability to their leaner subjects and different procedure, with measurement sites marked using a surgical marker and duplicate readings done on the same day, in a rotational order [6]. Except for marking the anatomical sites, our experimental protocol was similar to theirs, but the reliability of our measurements did not compare to theirs. We chose not to mark the measurement sites because in everyday practice it is unlikely that the subjects would maintain the markings from one test to another (typically taken weeks apart when %BF is tracked during a nutritional or lifestyle intervention).

Our study points out examiner performance as a possible cause of the difference in reliability between Wagner et al. [6] and other works. The reliability of T2 was higher than that of T1, suggesting that, unlike in the case of the BOD POD [34], US assessments of body composition do depend on the technician’s performance. This conclusion is supported also by the intertester bias, of about −0.5% BF, and the statistically significant differences between the mean subcutaneous adipose tissue thicknesses recorded by the two testers.

In rehabilitative US imaging, it is known that the precision of US-based morphometry hinges on the examiner’s ability to exert a consistent inward force on the transducer [35, 36]. The precision improved when the transducer was pressed against the skin by a constant-force spring [35], or when manual scanning was guided by a force-feedback device [37]. In our study, both testers had about the same experience with the BodyMetrix instrument (≈ 1 year); T1, however, also had 2 years of practice as a clinical sonographer, being used to exert higher axial forces on the transducer. Indeed, diagnostic US examinations often require forces of 5–14 N to rearrange superficial anatomic structures that impede the visualization of deeper ones [38]. Although our examiners were trained to follow the instructions of the instrument’s manufacturer [24], trying to exert a small, constant axial force (≈ 1 N) [35], they obtained significantly different results.

The limitations of this study include the use of automatic measurements and not blinding the testers to each other’s results. Although the BodyView software enables the examiner to override the automatic selection of the fat-muscle boundary, we did not use this facility. It might have provided, perhaps, an even better reliability. In this study, the tester was relieved of judging a result and focussed on assuring proper conditions for the automatic measurement. For logistic reasons, one tester served as recorder for the other. We assumed they would not influence each other if they record the output of automatic measurements in different regions of a spreadsheet. Looking at each other’s results would have required a conscious effort and they were trained not to do so. The observed intertester bias and reliability indices within the range reported in the literature suggest that they did not sway each other. Another limitation of this work is the sample size; it is large enough for studying each gender in part, but not for further stratifications (e.g., by age or nutritional status).

In conclusion, the statistical analysis of triplicate trials performed by two testers led to the inference that body composition assessments via A-mode US are not affected by learning effects. The intratester reliability was excellent for both testers, similar to that of ADP. Intertester reliability was very good, marginally smaller than its intratester counterpart. The precision of ultrasound assessments of percent body fat was slightly higher for men than for women. Hence, A-mode ultrasound is a portable, affordable, and reliable technique of body composition analysis. Therefore, it is a promising tool for bedside and event-site evaluation of nutritional status. Although its validity remains to be established for various populations, it is suitable for longitudinal studies that are more concerned with changes in body composition parameters than with their absolute values.

We observed an intertester bias and better indices of reliability for one of the examiners, presumably due to a smaller and more consistent axial force applied on the transducer. Future research will be needed to test whether force feedback will boost the reliability of body composition assessments via A-mode US, leading to consistent performance regardless of the examiner’s level of experience.

## Supplementary information

Supplementary Material
