# Supplementary material for: Reliability of body composition assessment using A-mode ultrasound in a heterogeneous sample
Source: Eur J Clin Nutr. 2020 Sep 11;75(3):438–45. doi: 10.1038/s41430-020-00743-y (PMC7943421; doi:10.1038/s41430-020-00743-y)
Supplement: Supplementary file 1 — Supplementary Material [file 41430_2020_743_MOESM1_ESM.docx]

**Supplementary Material**

**related to the paper**

**Reliability of body composition assessment using**

**A-mode ultrasound in a heterogeneous sample**

Monica Miclos-Balica^1^, Paul Muntean^1^, Falk Schick^1^, Horia G. Haragus^2^, Bogdan Glisici^1^, Vasile Pupazan^1^, Adrian Neagu^1,3^, Monica Neagu^1^

^1^Center for Modeling Biological Systems and Data Analysis, Department of Functional Sciences, Victor Babeş University of Medicine and Pharmacy Timişoara, Romania

^2^Department of Orthopedics, Victor Babeş University of Medicine and Pharmacy Timişoara, Romania

^3^Department of Physics & Astronomy, University of Missouri, Columbia, MO, U.S.A.

This study evaluates the intratester and intertester reliability of A-mode ultrasound for assessing human body composition. The results of triplicate trials performed by two testers were analyzed using statistical methods that characterize reliability. Percent body fat (%BF) assessments were based on 4 different formulas (7-sites Jackson and Pollock (JP7), 3-sites Jackson and Pollock (JP3), 3-sites Pollock (P3), and 1-point biceps (BIC)), implemented in the BodyView™ software shipped with the ultrasound instrument used in this study ˗ BodyMetrix™ BX2000 (Intelametrix, Livermore, CA, USA). This text presents the results that did not fit in the main article due to size limitations.

# **1. Intratester reliability**

The Bland-Altman (BA) plots depicted in Figure 1 of the main paper were obtained from the first two trials of Tester 1 (T1). Shown in Figure S1 are the corresponding BA plots of the first two trials of Tester 2 (T2).


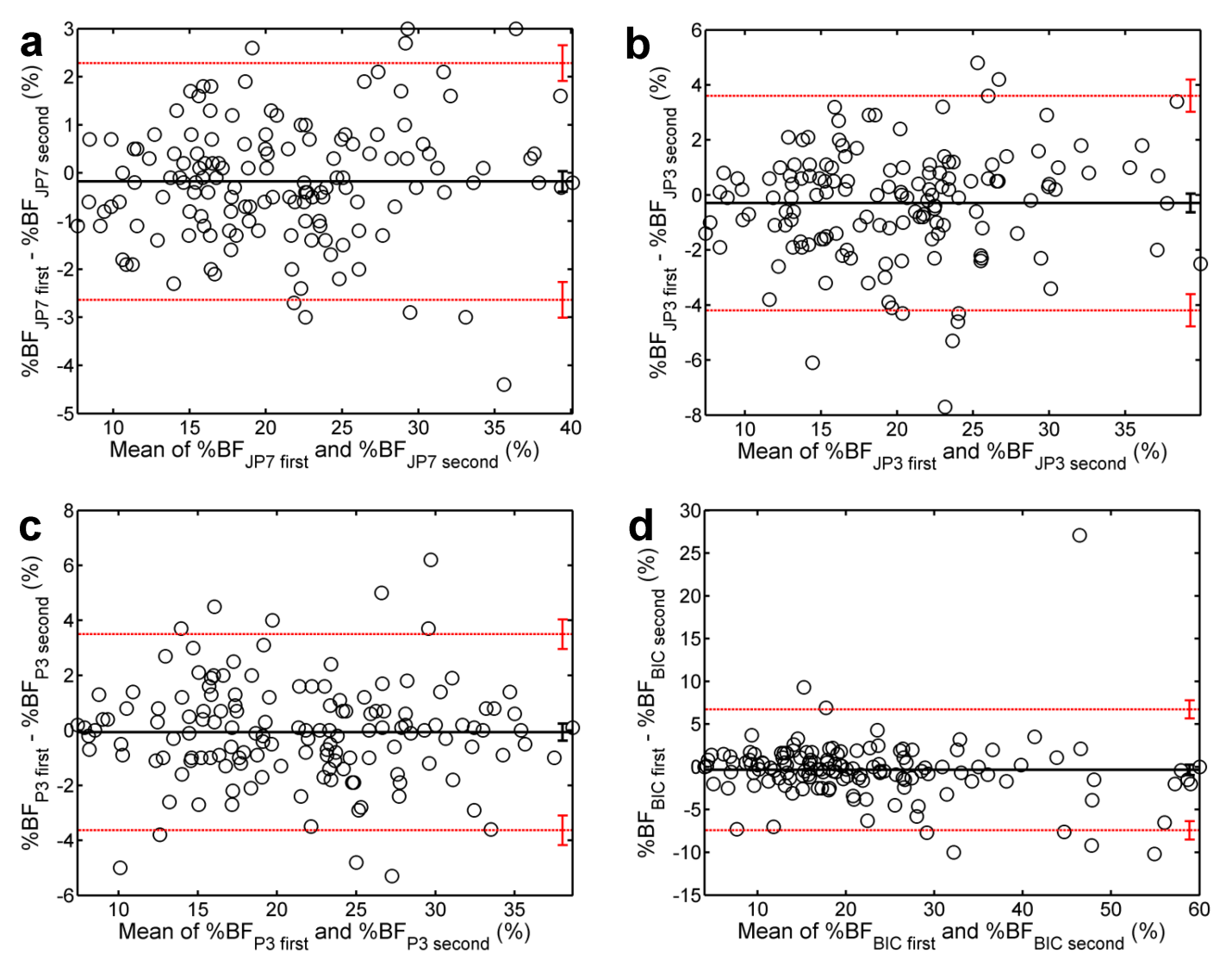


**Figure S1.** BA plots of differences vs. means of the first pair of %BF assessments performed by Tester 2 (T2) using various formulas: (a) 7-sites Jackson and Pollock (JP7), (b) 3-sites Jackson and Pollock (JP3), (c) 3-sites Pollock (P3), and (d) 1-point biceps (BIC). The thick horizontal line represents the bias, whereas the thin dotted lines represent the limits of agreement. The 95% confidence intervals (CI) of the bias and the limits of agreement are represented by error bars (vertical segments) on the right side of the plot.

Each point of such a BA plot corresponds to a pair of trials conducted on one subject and the horizontal axis indicates the mean of that pair of %BF assessments.

The 95% confidence intervals (CI) are represented in our BA plots by error bars crossing the lines that depict the bias (thick solid line) and the 95% limits of agreement (thin dotted lines). Note that the bias and 95% limits of agreement refer to a given sample. The 95% CI specifies the range of values of the corresponding quantity (e.g. bias) if other samples of the same size (n = 144) would be extracted randomly from the entire population.

Except for the results given by the BIC formula (panel (d)), the 95% intervals of agreement depicted in Figure S1 are slightly narrower than those of Figure 1 (main paper), indicating that T2 was more successful than T1 in replicating his own readings. This conclusion is supported by computing the differences ULA-LLA = 2×(ULA-Bias) for all pairs of trials (see Table S1).

**Table S1.** The results of BA analyses performed for three pairs of measurements (1-2, 1-3, and 2-3) done by two testers (Tester 1 and Tester 2), for %BF calculated using various formulas (JP7, JP3, P3, and BIC).

|  |  | **Tester 1** | | | | **Tester 2** | | | |
| --- | --- | --- | --- | --- | --- | --- | --- | --- | --- |
| **Formula** | **Pair** | **Bias^a^** | **95% CI** | **ULA** | **95% CI** | **Bias** | **95% CI** | **ULA** | **95% CI** |
| **JP7** | 1-2 | -0.11 | [-0.37, 0.14] | 2.84 | [2.40, 3.29] | -0.18 | [-0.39, 0.04] | 2.28 | [1.91, 2.65] |
|  | 1-3 | -0.04 | [-0.34, 0.25] | 3.33 | [2.82, 3.84] | -0.24 | [-0.45,-0.02] | 2.27 | [1.89, 2.65] |
|  | 2-3 | 0.07 | [-0.17, 0.31] | 2.80 | [2.39, 3.21] | -0.06 | [-0.29, 0.17] | 2.63 | [2.23, 3.04] |
| **JP3** | 1-2 | 0.03 | [-0.34, 0.40] | 4.26 | [3.62, 4.90] | -0.30 | [-0.63, 0.04] | 3.60 | [3.02, 4.19] |
|  | 1-3 | 0.03 | [-0.41, 0.46] | 5.02 | [4.26, 5.77] | -0.33 | [-0.66, 0.01] | 3.49 | [2.91, 4.06] |
|  | 2-3 | 0.00 | [-0.36, 0.36] | 4.10 | [3.48, 4.71] | -0.03 | [-0.40, 0.34] | 4.20 | [3.56, 4.84] |
| **P3** | 1-2 | -0.06 | [-0.44, 0.32] | 4.30 | [3.65, 4.96] | -0.07 | [-0.38, 0.24] | 3.50 | [2.96, 4.03] |
|  | 1-3 | -0.07 | [-0.45, 0.32] | 4.34 | [3.68, 5.01] | -0.23 | [-0.52, 0.07] | 3.17 | [2.66, 3.69] |
|  | 2-3 | -0.01 | [-0.37, 0.35] | 4.13 | [3.51, 4.76] | -0.16 | [-0.50, 0.18] | 3.71 | [3.13, 4.29] |
| **BIC** | 1-2 | -0.42 | [-1.04, 0.19] | 6.62 | [5.56, 7.68] | -0.36 | [-0.97, 0.26] | 6.72 | [5.65, 7.78] |
|  | 1-3 | -0.21 | [-0.64, 0.22] | 4.72 | [3.98, 5.46] | -0.03 | [-0.56, 0.50] | 6.07 | [5.15, 6.99] |
|  | 2-3 | 0.21 | [-0.39, 0.82] | 7.17 | [6.13, 8.22] | 0.33 | [-0.29, 0.94] | 7.40 | [6.34, 8.47] |

^a^ Bias, ULA and the limits of the 95% CI are expressed in the same units as the measured quantity (%BF).

The next two figures investigate the impact of gender on the intratester reliability of T2. The BA plots shown in Figures S2 and S3 were obtained for the first pair of trials completed by men and women, respectively.


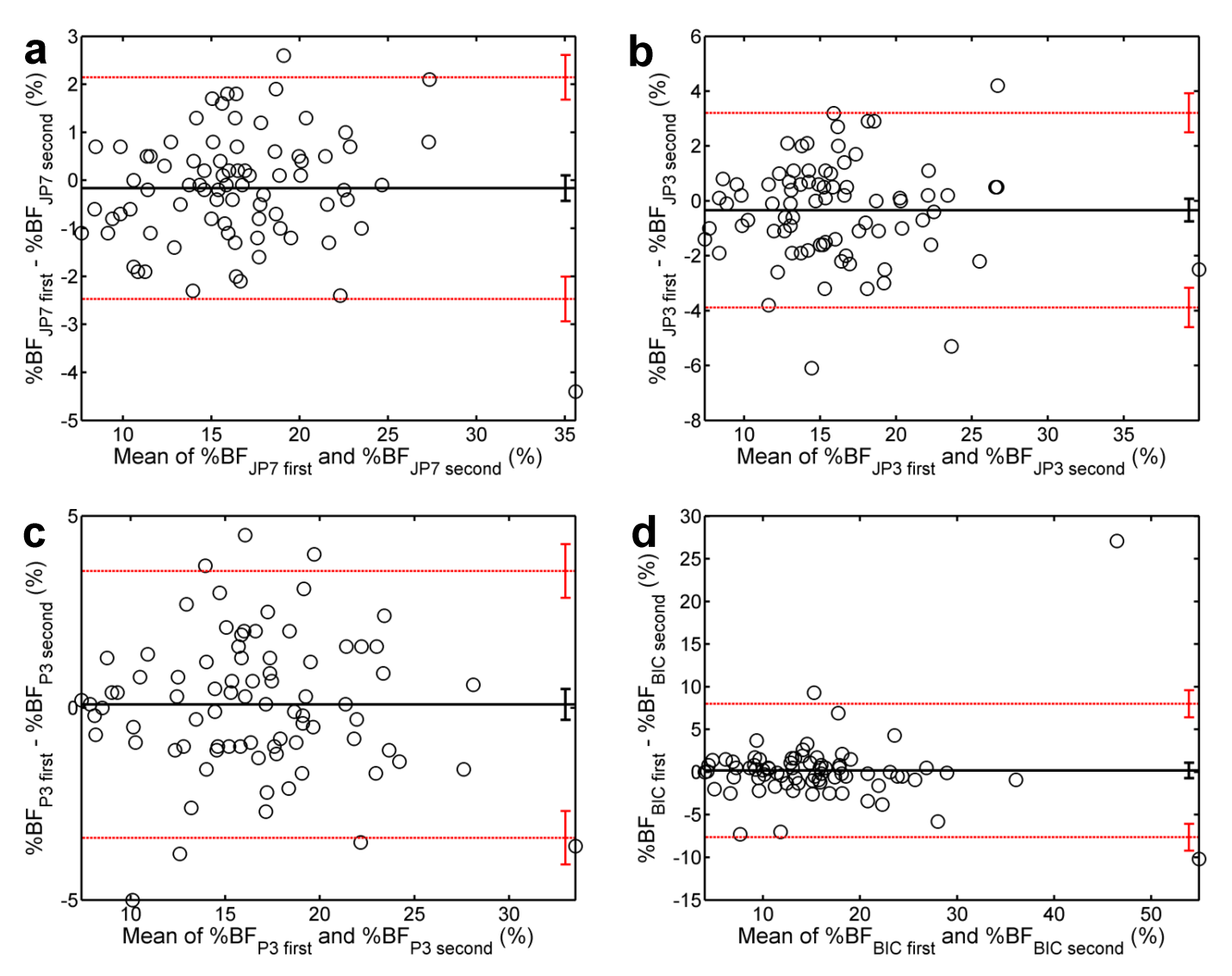


**Figure S2.** BA plots of the first pair of trials conducted by T2 on men using various prediction formulas for computing %BF: (a) JP7, (b) JP3, (c) P3, and (d) BIC. (Notations are explained in the caption of Figure S1.)

Note that the 95% CIs are narrower in Figure S1 than in Figures S2 and S3 mainly because Figure S1 refers to a larger sample.


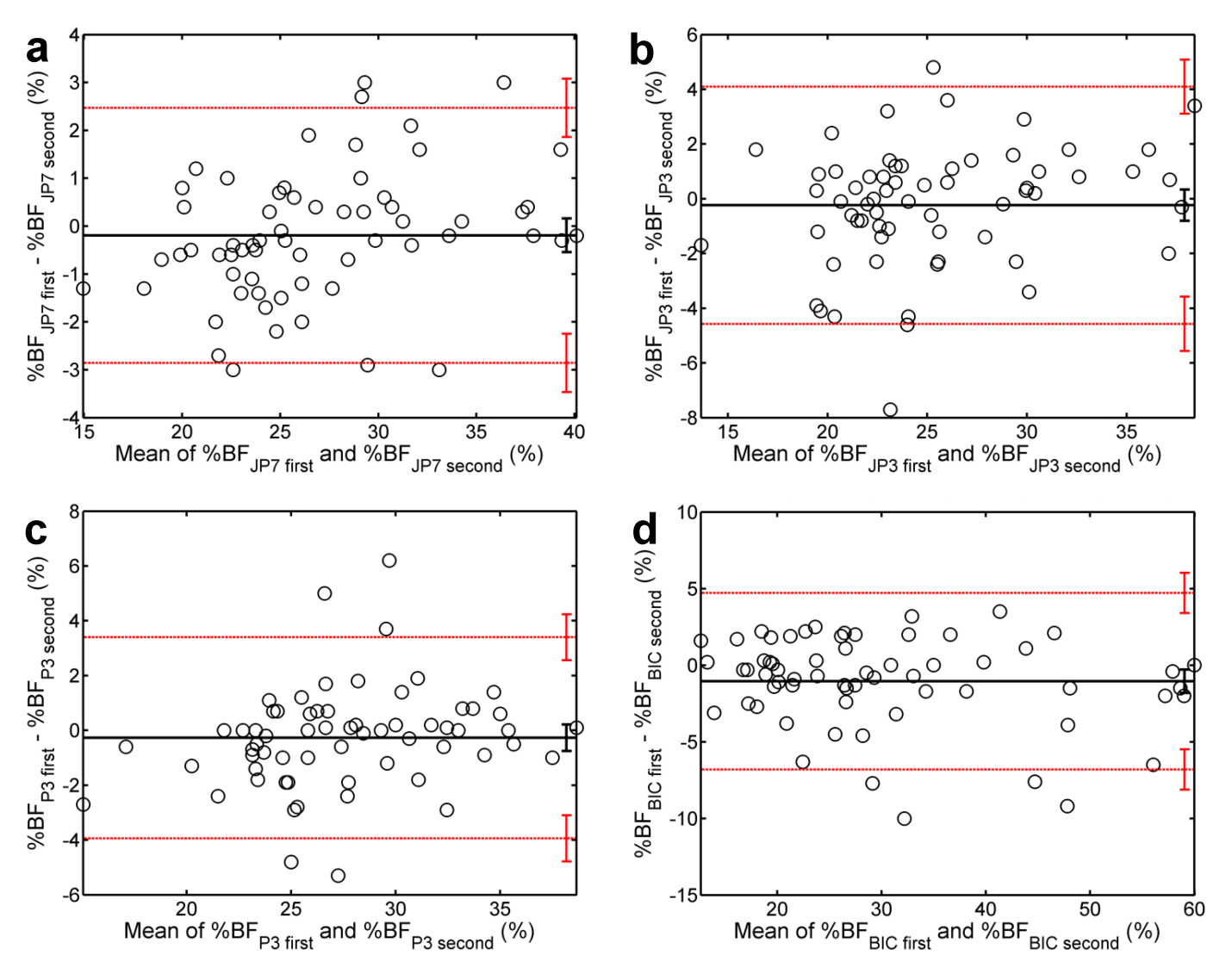


**Figure S3.** BA analysis of the first pair of trials conducted by T2 on women using different formulas for computing %BF: (a) JP7, (b) JP3, (c) P3, and (d) BIC.

Aside from the BIC formula, the 95% intervals of agreement are wider for women (e.g. in the context of the JP7 formula, ULA -LLA is 4.62 % BF for men and 5.32 % BF for women). When the BIC formula was used in the case of men (Figure S2d), T2 was more reliable for slim subjects than for subjects of high %BF (see the data points depicted on the right side of Figure S2d). The other formulas, however, did not point out a dependence of the %BF measurement precision on the subject's adiposity. A future study will be needed, involving at least 50 normal weight and 50 obese volunteers, to test whether the precision of US measurements is similar for these categories of subjects.

# **2. Intertester reliability**

The BA analysis of the intertester agreement between %BF values derived from the JP7 and JP3 formulas is described in the main paper (Figure 2). The corresponding analysis performed for the P3 and BIC formulas is represented in Figure S4. The P3 formula has led to the intertester bias of -0.54% BF and the width of the 95% interval of agreement, ULA - LLA = 8.86% BF (Figure S4a), demonstrating a close similarity with the JP3 formula (Figure 2b). The BIC formula led to a smaller bias (-0.29% BF), but the significantly wider 95% interval of agreement (ULA - LLA = 14 %) shows that intertester reliability was the poorest in this case (Figure S4b).


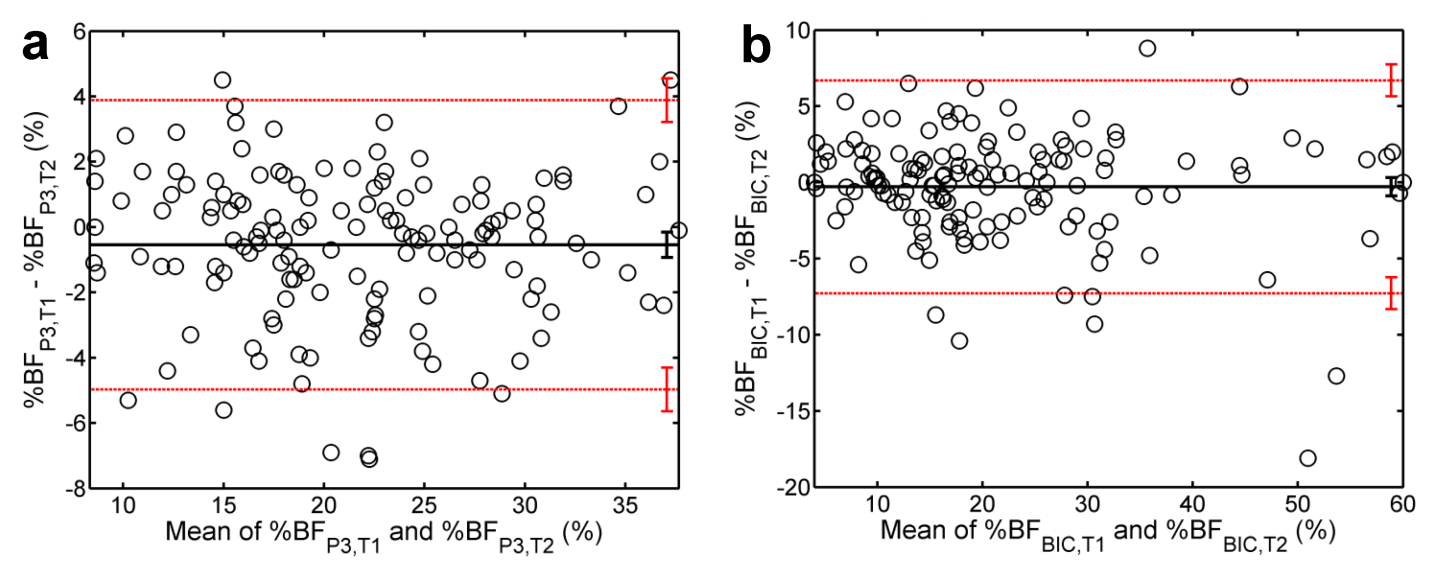


**Figure S4.** BA analysis of intertester agreement between %BF measurements based on (a) the P3 formula and (b) the BIC formula. Each plot represents differences vs. means of the first reading of Tester 1 (T1) and the third reading of Tester 2 (T2).

Figure S5 illustrates the gender dependence of the intertester reliability of %BF assessments using US measurements and the JP7 formula. The BA plots of Figure S5 reveal an intertester bias of -0.48% BF for men and -0.54% BF for women.


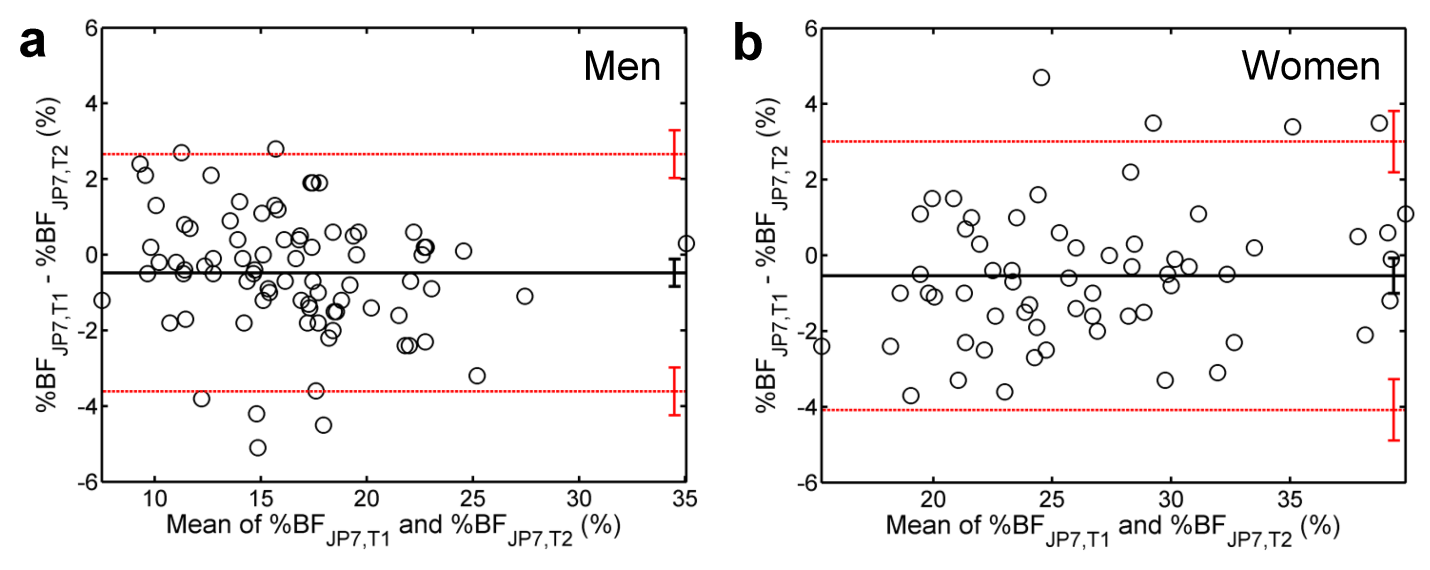


**Figure S5.** BA analysis of intertester agreement between %BF assessments based on the JP7 formula for (a) men and (b) women. Shown are differences vs. means of the first measurement of T1 and the third measurement of T2.

The bias is not the only indicator of the poorer intertester reliability observed in the case of women; the width of the 95% interval of agreement was 6.3% BF for men and 7.1% BF for women.

It is unclear why %BF measurements via US are less precise for women than for men. Possible reasons include the higher adiposity of the female body and the more challenging anatomic environment of certain sites considered in the prediction formulas. To elucidate this question, further studies will be needed that will explore various US instruments and prediction formulas.
